# Computational image analysis of distortion, sharpness, and depth of field in a next-generation hybrid exoscopic and microsurgical operative platform

**DOI:** 10.3389/fsurg.2024.1418679

**Published:** 2024-06-25

**Authors:** Wonhyoung Park, Irakliy Abramov, Thomas J. On, Yuan Xu, Andrea L. Castillo, Nicolas I. Gonzalez-Romo, Roland Guckler, Mark C. Preul

**Affiliations:** ^1^ The Loyal and Edith Davis Neurosurgical Research Laboratory, Department of Neurosurgery, Barrow Neurological Institute, St. Joseph's Hospital and Medical Center, Phoenix, AZ, United States; ^2^ Carl Zeiss Meditec AG, Jena, Oberkochen, Germany

**Keywords:** camera optics, computer vision, depth of field, exoscopic surgery, machine learning, mathematical algorithms, operating microscope

## Abstract

**Objective:**

The development of surgical microscope-associated cameras has given rise to a new operating style embodied by hybrid microsurgical and exoscopic operative systems. These platforms utilize specialized camera systems to visualize cranial neuroanatomy at various depths. Our study aims to understand how different camera settings in a novel hybrid exoscope system influence image quality in the context of neurosurgical procedures.

**Methods:**

We built an image database using captured cadaveric dissection images obtained with a prototype version of a hybrid (microsurgical/exoscopic) operative platform. We performed comprehensive 4K-resolution image capture using 76 camera settings across three magnification levels and two working distances. Computer algorithms such as structural similarity (SSIM) and mean squared error (MSE) were used to measure image distortion across different camera settings. We utilized a Laplacian filter to compute the overall sharpness of the acquired images. Additionally, a monocular depth estimation deep learning model was used to examine the image's capability to visualize the depth of deeper structures accurately.

**Results:**

A total of 1,368 high-resolution pictures were captured. The SSIM index ranged from 0.63 to 0.85. The MSE was nearly zero for all image batches. It was determined that the exoscope could accurately detect both the sharpness and depth based on the Laplacian filter and depth maps, respectively. Our findings demonstrate that users can utilize the full range of camera settings available on the exoscope, including adjustments to aperture, color saturation, contrast, sharpness, and brilliance, without introducing significant image distortions relative to the standard mode.

**Conclusion:**

The evolution of the camera incorporated into a surgical microscope enables exoscopic visualization during cranial base surgery. Our result should encourage surgeons to take full advantage of the exoscope's extensive range of camera settings to match their personal preferences or specific clinical requirements of the surgical scenario. This places the exoscope as an invaluable asset in contemporary surgical practice, merging high-definition imaging with ergonomic design and adaptable operability.

## 1 Introduction

Neurosurgery requires sharp and clear operative visualization to perform surgical maneuvers safely near critical neurovascular structures. Current generations of microscopes and endoscopes provide high-resolution magnification of cranial targets to neurosurgeons, enabling refined exposures through deep and narrow operative corridors. The operative view provided by the microscope is familiar to neurosurgeons early in their residency training. Vision through a surgical microscope is stereoscopic and binocular. The image is a product of the reflection of light that travels through a specialized system of sequential lenses and tubes (1). These instruments have become a modern standard of care (2).

In recent years, operating exoscope systems have emerged as an alternate visualization method (3), implementing specialized three-dimensional (3D) camera devices that orbit the surgical field at potentially greater distances than operating microscopes (4–6). The 3D image that the surgeon observes in the display is a composition of images with a minor offset to provide a sense of depth. This 3D image can be modified, enhanced, and augmented digitally to offer an optimized view of the surgical field (4). In exoscopic surgery, the operative view is streamed to a large 3D monitor that the entire surgical team can observe (3, 4, 7–8). Reported advantages of exoscopic systems over traditional surgical microscopes include improved ergonomics and maneuverability for the surgeon, as well as improved synchronization and communication with the operating room staff via additional viewing screens (7, 8). Some disadvantages of exoscopic systems include a lack of familiarity, requiring surgeons to adapt to a heads-up display where they face a screen instead of viewing through microscope oculars. In addition, the operating room must be reorganized to ensure unobstructed sight lines to the monitors. Other challenges include difficulty assisting if not the primary surgeon, and the lack of imaging system movement, such as using microscope mouthpiece control (7–9).

This study evaluated a next-generation hybrid visualization system combining microsurgical and exoscopic functions with enhanced 4K camera capture and video recording capabilities. The platform is intended to set a new benchmark in surgical visualization, enabling surgeons to adjust the camera settings of the captured images based on personal preference or clinical situation. We aimed to quantitively measure through mathematical algorithms and deep learning how different camera settings influence image quality, depth, and edge detection in the context of various approaches and targets. This study is not a product endorsement but was performed on a prototype neurosurgical operating microscope-exoscope system combining these functions in one imaging head piece that supports various image capture modalities and enhancement or manipulation settings. Algorithms and methods for objectively evaluating operative neurosurgical digital image quality have yet to be well established. This study may be a touchstone for developing new tools and techniques specific to neurosurgical digital images.

## 2 Methods

### 2.1 Study design

**Figure 1:**
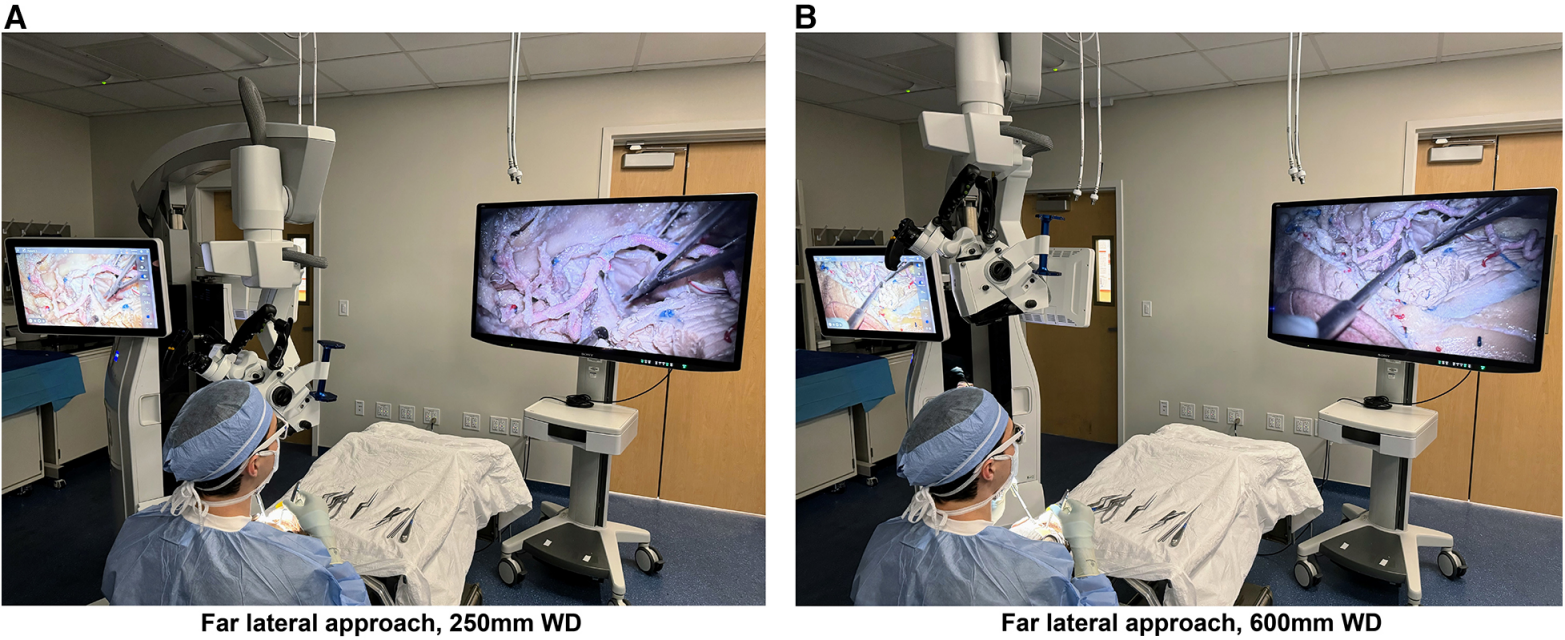
Photographs of the next-generation hybrid exoscopic and microsurgical operative platform, with the exoscope camera set to standard mode, being used at 250 mm (**A**) and 600 mm (**B**) working distances from the cadaveric specimen for a far lateral approach. The hybrid system is positioned on the left and the 3D screen on the right, with the user wearing 3D glasses. Used with permission from Barrow Neurological Institute, Phoenix, Arizona.

We performed a cadaveric dissection study using standard microsurgical instruments and a prototype of the next-generation KINEVO 900 robotic visualization system (Carl Zeiss Meditec AG, Oberkochen, Germany) (Figure 1). This hybrid system combines surgical microscopical and exoscopic functions in one head piece with enhanced 4K camera capture and video recording capabilities. A 55″ three-dimensional display (LMD-X550MT monitor, Sony Corp., Tokyo, Japan) was used for 3D visualization through polarized 3D glasses.

### 2.2 Surgical procedure and image assessment

Preserved cadaveric latex-injected heads (red for arteries, blue for veins) were dissected (by four experienced neurosurgeons: WP, IA, YX, NIGR) using standard neurosurgical instruments and cranial fixation using a Mayfield clamp device. Surgical approaches exposed the structures of interest for image assessment. An orbitozygomatic approach was performed, followed by the Sylvian fissure split to expose the M2 trunks of the middle cerebral artery (MCA). The temporal pole was retracted to expose the basilar artery apex through the carotid-oculomotor window. A far lateral approach was performed to expose the posterior inferior cerebellar artery (PICA) origin at the V4 segment. Images were acquired using the laser overlay for determination by using two different working distances from three different anatomical targets, at working distances of 250 mm and 600 mm. Low, medium, or high magnification setting was used at each different working distance and anatomical target.

The role of different image modalities on image distortion, depth, and edge detection was analyzed, using reference original images from standard mode for comparison. In the surgical field, numbered tags were assigned to each of the 76 distinct camera settings and positioned identically for each set of working distance and magnification. These camera settings were carefully selected to showcase the potential setting variations that neurosurgeons may utilize in clinical practice. We tested different camera modes, including standard, depth, and custom modes, as well as adjustments to aperture, contrast, sharpness, color saturation, and brilliance.

### 2.3 Computer analysis of surgical pictures

Using Python 3.8 interpreter, the scikit-image library was used to calculate image quality metrics, including the structural similarity index (SSIM) and mean squared error (MSE). These algorithms are signal fidelity measurements (10) used to compare the level of distortion between images. SSIM is designed to identify similarities between pixels in different images. SSIM score is a measure ranging from −1 to 1, reflecting the similarity between two images. Values closer to 1 indicate a higher similarity. In contrast, MSE quantifies the differences between pixels within images. MSE scores range from 0 to infinity. A value of 0 indicates no difference from the original image, and values increasing from 0 signify greater deviation from the original image.

### 2.4 Edge detection using a Laplacian filter

The OpenCV computer vision library was used to implement a Laplacian filter. This is an image operator or kernel that can be used to quantify the sharpness of an image by measuring regions of rapid pixel intensity changes, such as edges and fine details. The dispersion or variance of the filter measurement provides an estimate of the amount of high-frequency content or sharpness present in an image. Higher values in the Laplacian variance output indicate an image with pronounced edge definition and greater sharpness. In contrast, lower values point to a smoother image, characterized by more blurriness and less sharpness. However, the Laplacian filter variance output is relative to a specific image, and a lower Laplacian filter output may still have relatively good edge detection. Therefore, assessing the Laplacian filter image generated for each camera setting was also necessary.

### 2.5 Monocular depth estimation

A deep learning model trained to calculate pixel-level depth from 2D images was used to compute depth maps and histogram plots of depth allocations by performing image recognition of depth cues present in the image. A vision transformer neural network (MiDaS) was used to estimate depth allocations by measuring pixel frequency variance. This model measures depth perception in 2D images based on different camera settings. The generated depth maps were analyzed based on color to assess depth detection. Purple colors represented deeper structures, and yellow colors represented superficial structures.

## 3 Results

### 3.1 Descriptive analysis

**Figure 2:**
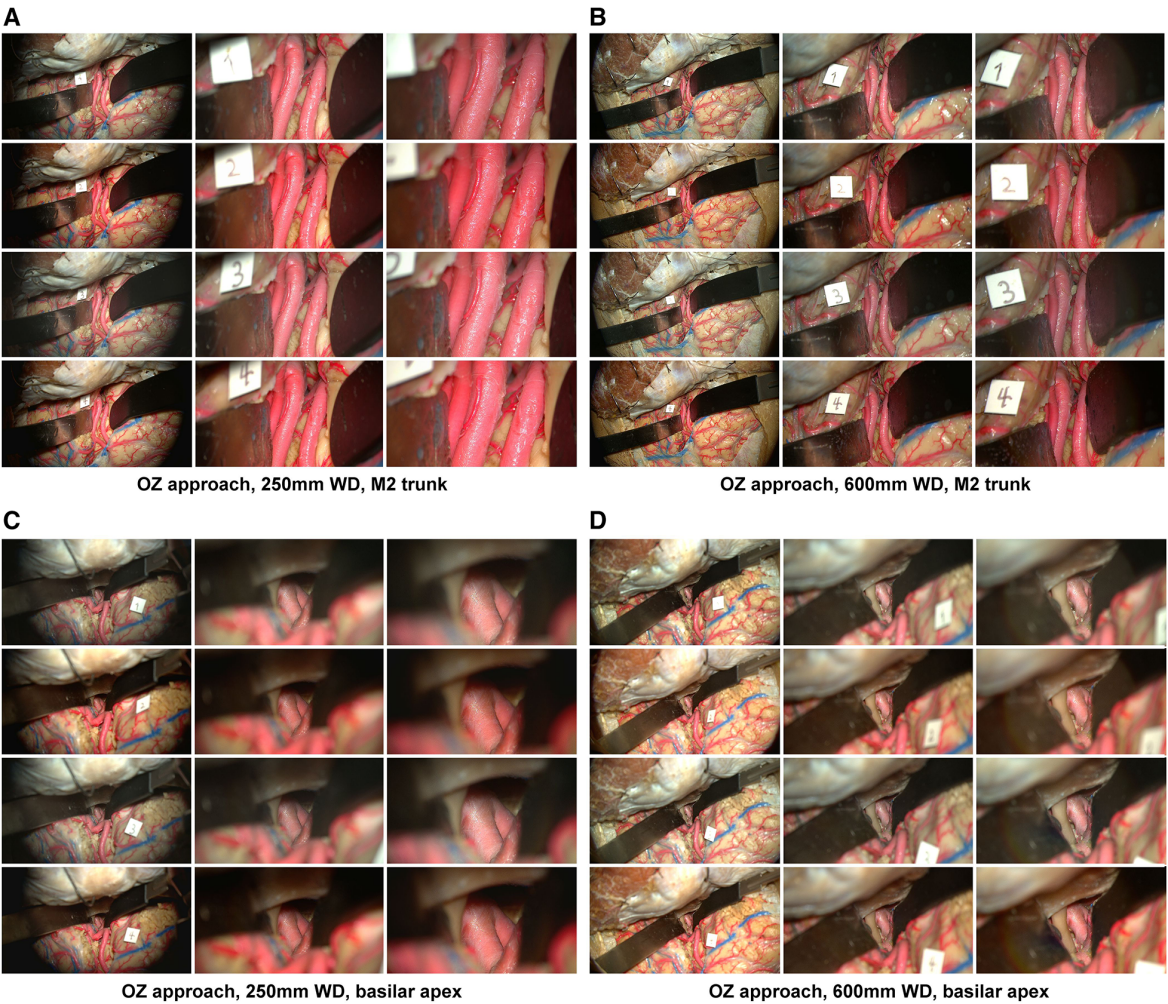
Image database, orbitozygomatic approach. Columns depict low, intermediate, and high-magnification images. Rows correspond to different camera settings, 1: standard mode, 2: standard mode with brilliance mode on, 3: depth mode, 4: depth mode with brilliance mode on. (**A**) Acquisition of images using a 250 mm working distance. The Sylvian fissure was dissected, and the M2 trunks were exposed over the insula. (**B**) A 600 mm working distance is used to capture images of the same approach. (**C**) Temporopolar exposure of the basilar apex using the carotid-oculomotor window. Images were captured using a 250 mm working distance. (**D**) The working distance was increased to 600 mm to capture images of the same approach. Used with permission from Barrow Neurological Institute, Phoenix, Arizona.

**Figure 3:**
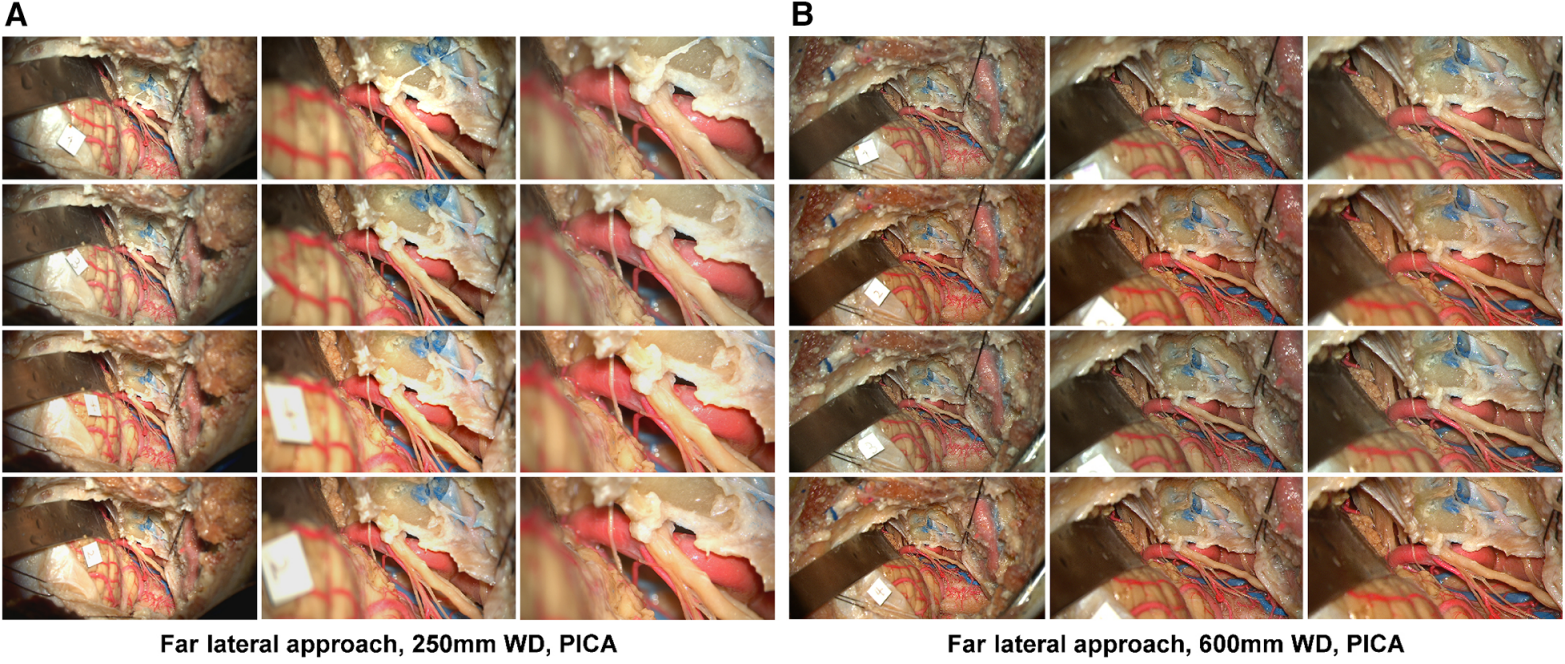
Image database, far lateral approach. Columns depict low, intermediate, and high-magnification images. Rows correspond to the image labels 1: standard mode, 2: standard mode brilliance on, 3: depth mode, 4: depth mode brilliance on. Acquisition of images using a 250 mm working distance (**A**) and 650 mm working distance (**B**) The accessory nerve is seen coursing towards the jugular foramen. The PICA branches off the V4 segment. Used with permission from Barrow Neurological Institute, Phoenix, Arizona.

1,368 high-resolution cadaveric dissection pictures were captured (Figures 2, 3). For each image, image distortion was measured with SSIM and MSE scores relative to a reference image from the standard mode. A Laplacian-filtered image and a depth map were also created, for a total of 4,104 images in the database. Continuous variables were reported as mean (SD). There were two different working distances, 250 mm and 600 mm. For each working distance, three magnification settings were tested: low, medium, and high. 76 images were taken for each working distance and magnification combination. This method was tested on three neurosurgical approaches: orbitozygomatic temporopolar, orbitozygomatic transsylvian, far lateral transcondylar.

### 3.2 Orbitozygomatic transsylvian approach

#### 3.2.1 SSIM and MSE scores

At 250 mm working distance, the SSIM score was 0.74 (0.05), 0.71 (0.05), and 0.69 (0.06) for low, intermediate, and high magnification images, respectively (Table 1). At 600 mm working distance, the SSIM score was 0.66 (0.06), 0.77 (0.05), and 0.75 (0.07) for low, intermediate, and high magnification images, respectively.

**Table 1:** Image distortion analysis of exoscope camera images using various surgical approaches .

Surgical approach	Working distance	Target	Magnification	Images	SSIM Mean (SD)	MSE Mean (SD)
Orbitozygomatic transsylvian	250 mm	M2 trunks	Low	76	0.74 (0.05)	0.005 (0.001)
	250 mm	M2 trunks	Int	76	0.71 (0.05)	0.021 (0.006)
	250 mm	M2 trunks	High	76	0.69 (0.06)	0.026 (0.011)
	600 mm	M2 trunks	Low	76	0.66 (0.06)	0.009 (0.003)
	600 mm	M2 trunks	Int	76	0.77 (0.05)	0.011 (0.005)
	600 mm	M2 trunks	High	76	0.75 (0.07)	0.017 (0.009)
Orbitozygomatic temporopolar	250 mm	Basilar artery apex	Low	76	0.82 (0.04)	0.008 (0.002)
	250 mm	Basilar artery apex	Int	76	0.85 (0.05)	0.004 (0.003)
	250 mm	Basilar artery apex	High	76	0.84 (0.05)	0.003 (0.003)
	600 mm	Basilar artery apex	Low	76	0.78 (0.05)	0.011 (0.006)
	600 mm	Basilar artery apex	Int	76	0.82 (0.05)	0.011 (0.008)
	600 mm	Basilar artery apex	High	76	0.79 (0.08)	0.010 (0.012)
Far lateral transcondylar	250 mm	PICA (p1)	Low	76	0.63 (0.05)	0.025 (0.005)
	250 mm	PICA (p1)	Int	76	0.68 (0.05)	0.033 (0.007)
	250 mm	PICA (p1)	High	76	0.69 (0.05)	0.039 (0.016)
	600 mm	PICA (p1)	Low	76	0.66 (0.06)	0.009 (0.004)
	600 mm	PICA (p1)	Int	76	0.68 (0.05)	0.008 (0.002)
	600 mm	PICA (p1)	High	76	0.71 (0.05)	0.008 (0.005)

Higher SSIM and lower MSE scores indicate greater image detail preservation and lower distortion in comparison to a standard mode reference image.

At 250 mm working distance, the MSE score was 0.005 (0.001), 0.021 (0.006), and 0.026 (0.011) for low, intermediate, and high magnification images, respectively. At 600 mm working distance, the MSE score was 0.009 (0.003), 0.011 (0.005), and 0.017 (0.009) for low, intermediate, and high magnification images, respectively.

#### 3.2.2 Edge detection

**Figure 4:**
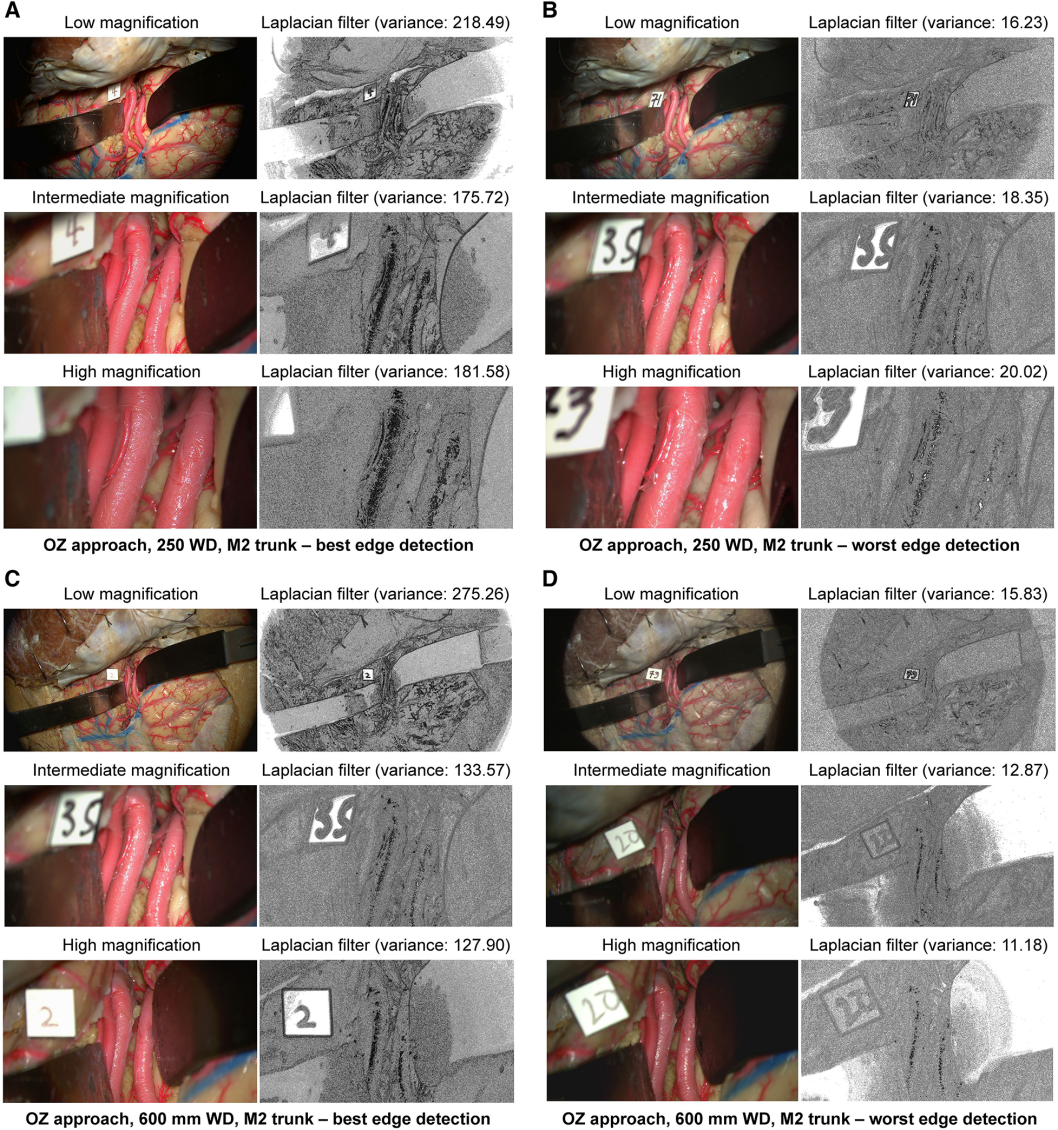
Image sharpness quantification of orbitozygomatic trans-sylvian exposures at different magnifications and working distances. Rows show low, intermediate, and high magnification images. Grayscale images represent the Laplacian filtered images, which measure edge detection through sharpness. (**A**) 250 mm working distance, best edge detection images. (**B**) 250 mm working distance, worse edge detection images. (**C**) 600 mm working distance, best edge detection images. (**D**) 600 mm working distance, worst edge detection images. Camera settings 2 and 4 often appeared in the best edge detection group coincidentally. Similar camera setting permutations also showed average or low Laplacian filter variance. Used with permission from Barrow Neurological Institute, Phoenix, Arizona.

At 250 mm working distance, the Laplacian filter variance was 80.1 (58.0), 63.1 (39.3), and 68.5 (42.9) for low, intermediate, and high magnification images, respectively. At 600 mm working distance, the Laplacian filter variance was 87.6 (66.7), 49.8 (32.3), and 46.2 (29.6) for low, intermediate, and high magnification images. The minimum and maximum Laplacian filter variance images for both working distances are reported in Figure 4.

#### 3.2.3 Depth estimation

**Figure 5:**
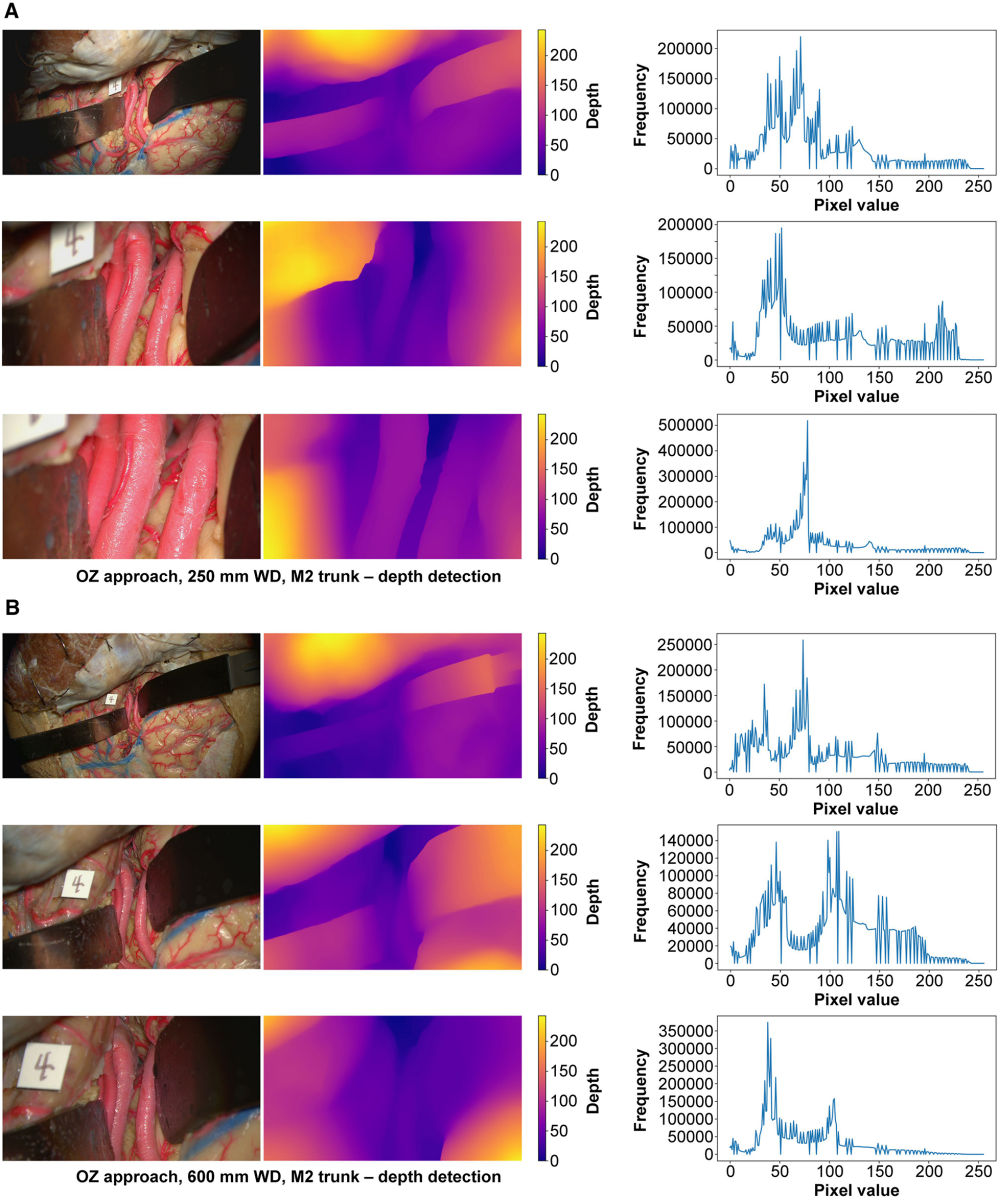
Monocular depth estimation. Orbitozygomatic approach, exposure of the M2 trunks. Rows correspond to low, intermediate, and high magnification images. Columns correspond to the original image (left), depth map (center), and histogram (right). (**A**) 250 mm working distance. (**B**) 600 mm working distance images. Used with permission from Barrow Neurological Institute, Phoenix, Arizona.

At 250 mm working distance, the depth map histogram pixel frequency variance was 1,413.8 (71.0), 1,344.1 (95.1), and 1,324.4 (120.9) for low, intermediate, and high magnification images, respectively (Figure 5). At 600 mm working distance, the depth map histogram pixel frequency variance was 1,365.9 (112.7), 1,317.9 (75.6), and 1,357.8 (40.7) for low, intermediate, and high magnification images, respectively.

### 3.3 Orbitozygomatic temporopolar approach

#### 3.3.1 SSIM and MSE scores

At 250 mm working distance, the SSIM score was 0.82 (0.04), 0.85 (0.05), and 0.84 (0.05) for low, intermediate, and high magnification images, respectively (Table 1). At 600 mm working distance, the SSIM score was 0.78 (0.05), 0.82 (0.05), and 0.79 (0.08) for low, intermediate, and high magnification images, respectively.

At 250 mm working distance, the MSE score was 0.008 (0.002), 0.004 (0.003), and 0.003 (0.003) for low, intermediate, and high magnification images, respectively. At 600 mm working distance, the MSE score was 0.011 (0.006), 0.011 (0.008), and 0.010 (0.012) for low, intermediate, and high magnification images, respectively.

#### 3.3.2 Edge detection

**Figure 6:**
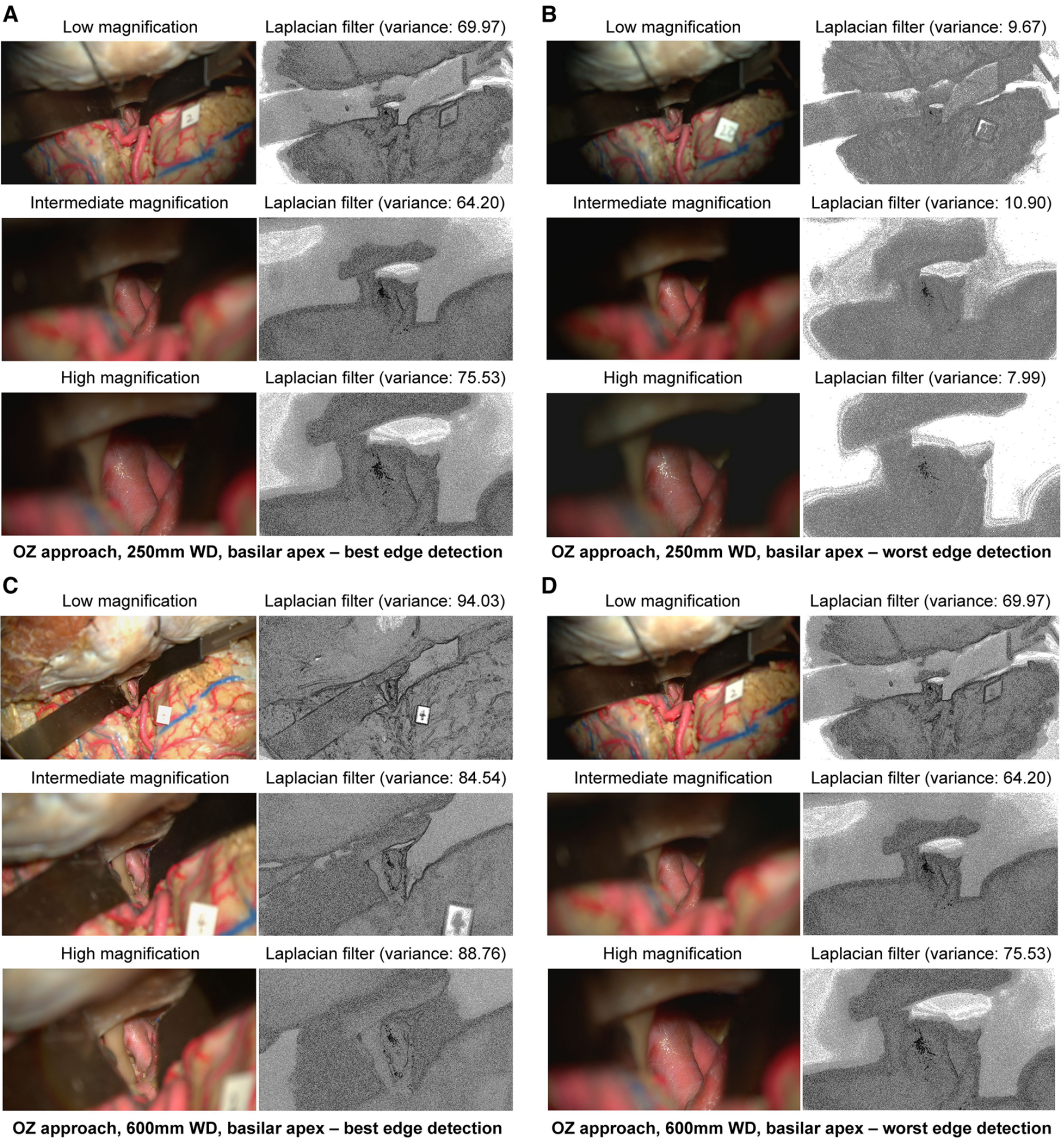
Image sharpness quantification of orbitozygomatic temporopolar basilar apex exposure at different magnifications and working distances. Rows show low, intermediate, and high magnification images. Grayscale images represent the Laplacian filtered images, which measure edge detection through sharpness. (**A**) 250 mm working distance, best edge detection images. (**B**) 250 mm working distance, worse edge detection images. (**C**) 600 mm working distance, best edge detection images. (**D**) 600 mm working distance, worst edge detection images. Used with permission from Barrow Neurological Institute, Phoenix, Arizona.

At 250 mm working distance, the Laplacian filter variance was 29.8 (18.0), 28.8 (15.3), and 29.3 (18.0) for low, intermediate, and high magnification images, respectively. At 600 mm working distance, the Laplacian filter variance was 42.5 (25.8), 34.8 (18.7), and 34.9 (19.9) for low, intermediate, and high magnification images, respectively. The minimum and maximum Laplacian filter variance images for both working distances are reported in Figure 6.

#### 3.3.3 Depth estimation

**Figure 7:**
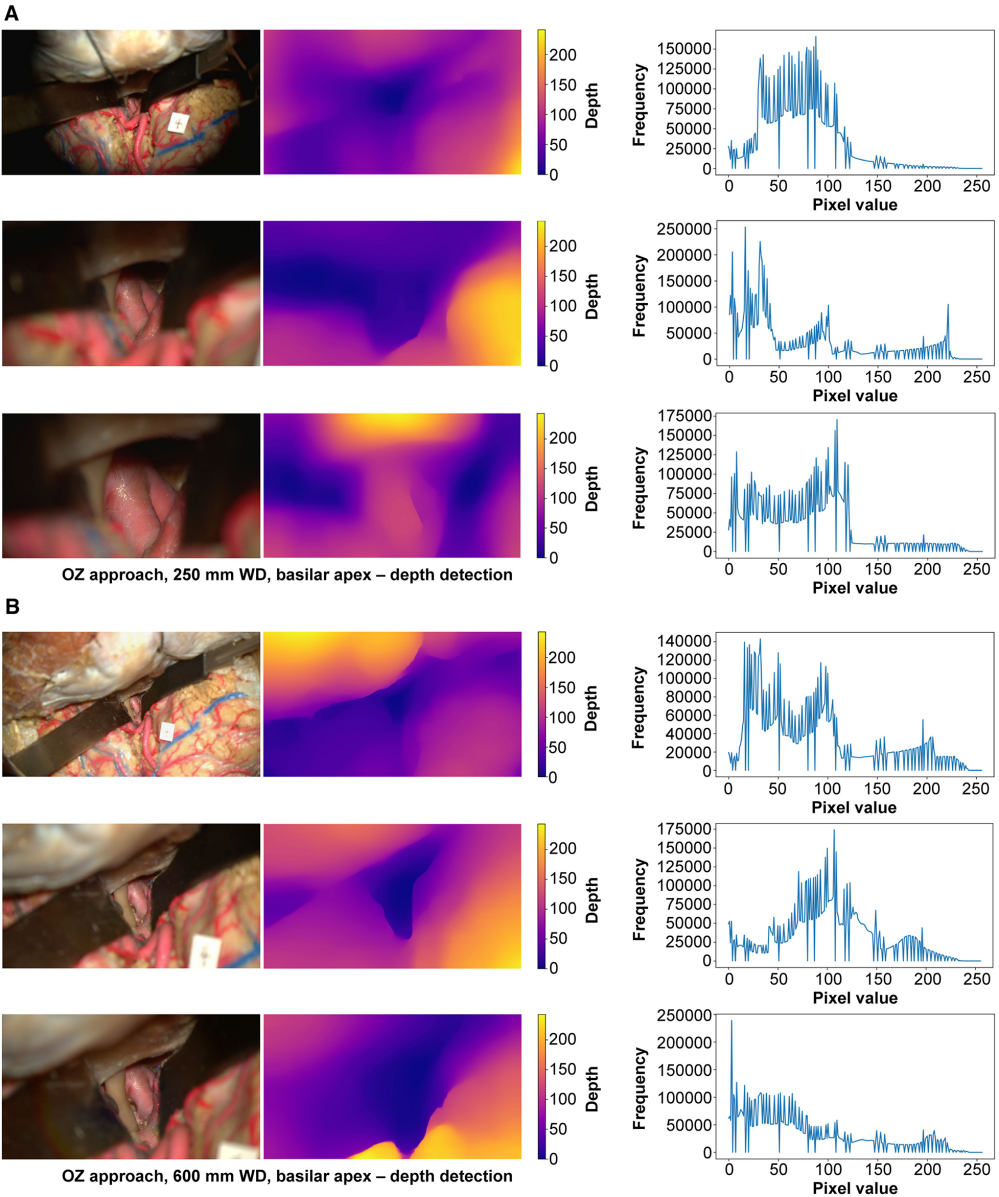
Monocular depth estimation. Orbitozygomatic approach, basilar apex exposure. Rows correspond to low, intermediate, and high magnification images. Columns correspond to the original image (left), depth map (center), and histogram (right). (**A**) 250 mm working distance. (**B**) 600 mm working distance images. Used with permission from Barrow Neurological Institute, Phoenix, Arizona.

At 250 mm working distance, the depth map histogram pixel frequency variance was 1,340.5 (78.5), 1,407.5 (92.7), and 1,427.6 (98.8) for low, intermediate, and high magnification images, respectively (Figure 7). At 600 mm working distance, the depth map histogram pixel frequency variance was 1,365.9 (112.7), 1,317.9 (75.6), and 1,357.8 (40.7) for low, intermediate, and high magnification images, respectively.

### 3.4 Far lateral approach

#### 3.4.1 SSIM and MSE scores

At 250 mm working distance, the SSIM score was 0.63 (0.05), 0.68 (0.05), and 0.69 (0.05) for low, intermediate, and high magnification images, respectively (Table 1). At 600 mm working distance, the SSIM score was 0.66 (0.06), 0.68 (0.05), and 0.71 (0.05) for low, intermediate, and high magnification images, respectively.

At 250 mm working distance, the MSE score was 0.025 (0.005), 0.033 (0.007), and 0.039 (0.016) for low, intermediate, and high magnification images, respectively. At 600 mm working distance, the MSE score was 0.009 (0.004), 0.008 (0.002), and 0.008 (0.005) for low, intermediate, and high magnification images, respectively.

#### 3.4.2 Edge detection

**Figure 8:**
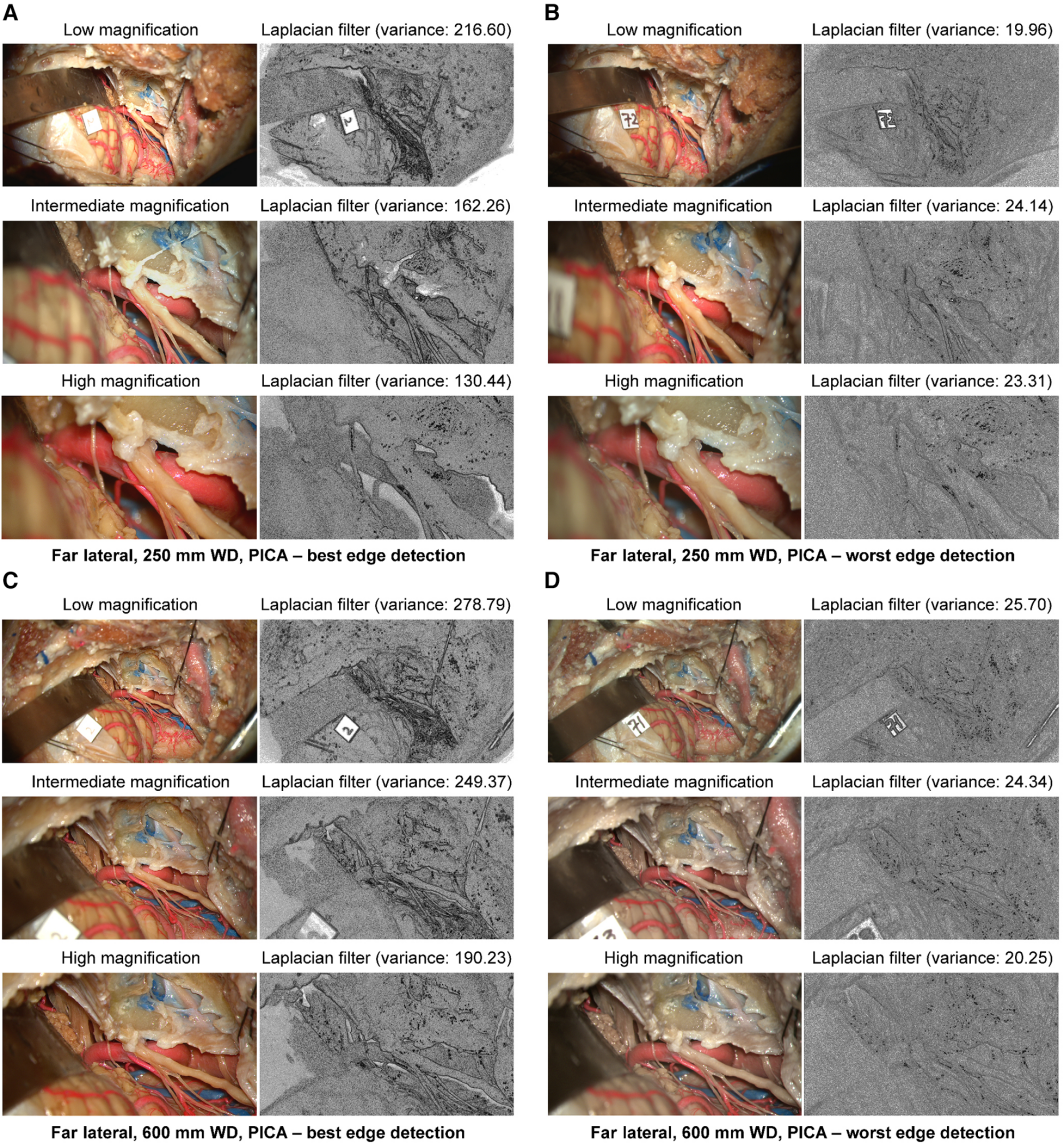
Image sharpness quantification of the far lateral exposure at different magnifications and working distances. Rows show low, intermediate, and high magnification images. Grayscale images represent the Laplacian filtered images, which measure edge detection through sharpness. (**A**) 250 mm working distance, best edge detection images. (**B**) 250 mm working distance, worse edge detection images. (**C**) 600 mm working distance, best edge detection images. (**D**) 600 mm working distance, worst edge detection images. Used with permission from Barrow Neurological Institute, Phoenix, Arizona.

At 250 mm working distance, the Laplacian filter variance was 89.2 (62.6), 76.1 (44.8), and 64.5 (35.5) for low, intermediate, and high magnification images, respectively. At 600 mm working distance, the Laplacian filter variance was 110.5 (75.7), 98.9 (66.2), and 82.3 (53.9) for low, intermediate, and high magnification images, respectively. The minimum and maximum Laplacian filter variance images for both working distances are reported in Figure 8.

#### 3.4.3 Depth estimation

**Figure 9:**
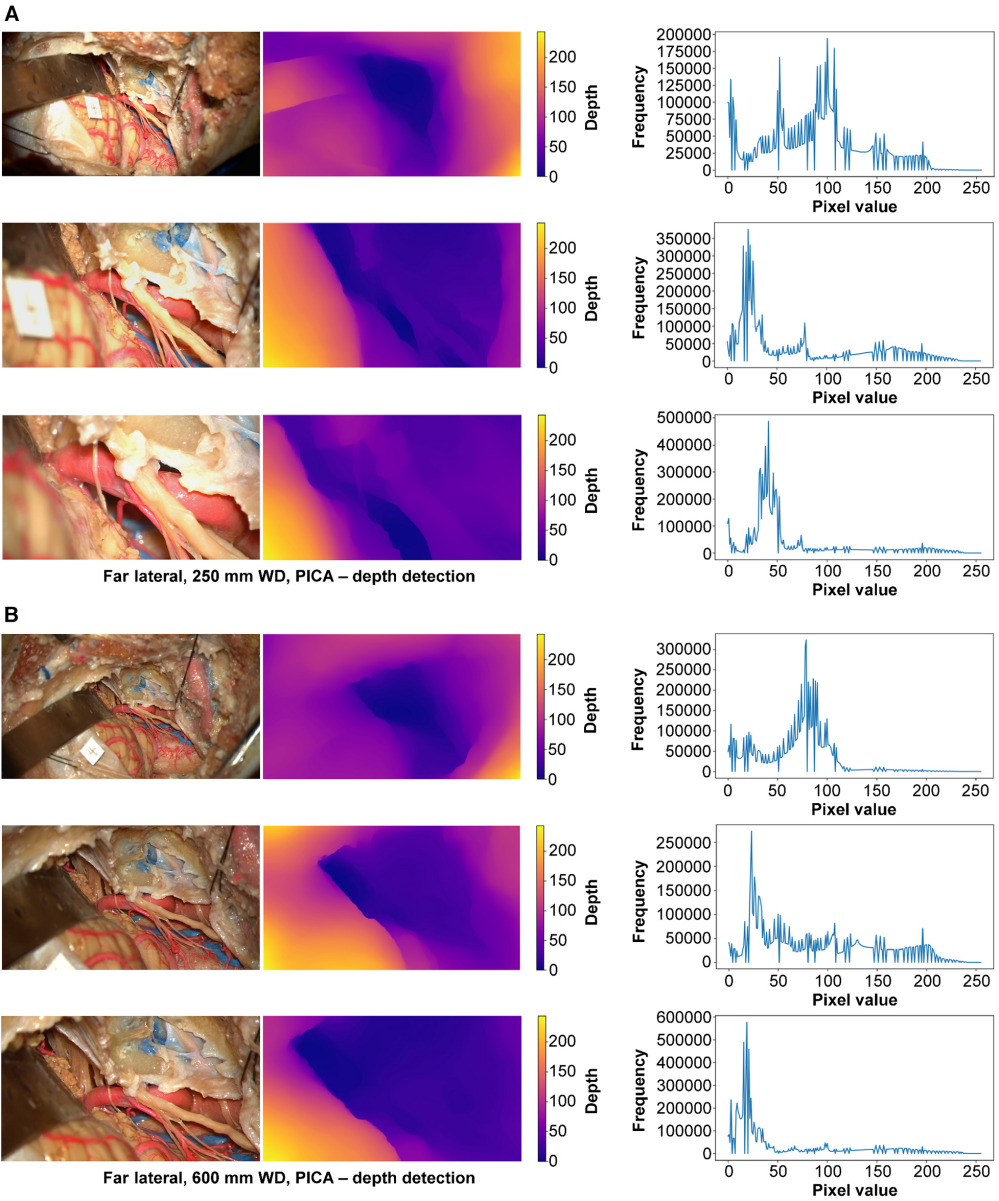
Monocular depth estimation. Far lateral exposure. Rows correspond to low, intermediate, and high magnification images. Columns correspond to the original image (left), depth map (center), and histogram (right). (**A**) 250 mm working distance. (**B**) 600 mm working distance images. Used with permission from Barrow Neurological Institute, Phoenix, Arizona.

At 250 mm working distance, the depth map histogram pixel frequency variance was 1,576.8 (92.2), 1,421.2 (53.1), and 1,565.0 (87.0) for low, intermediate, and high magnification images, respectively (Figure 9). At 600 mm working distance, the depth map histogram pixel frequency variance was 1,422.0 (104.5), 1,393.9 (51.2), and 1,675.2 (30.5) for low, intermediate, and high magnification images, respectively.

## 4 Discussion

When utilizing an exoscope in surgical settings, it is essential to recognize that this tool is distinct from a traditional microscope. An exoscope is a high-definition digital camera system offering unique functionalities tailored to modern surgical requirements. Unlike conventional microscopes, the exoscope's high-definition camera allows for various adjustable parameters, such as working distance, aperture, camera modes, and other image modifications. This flexibility in settings enables the exoscope to cater to specific visual needs, providing a level of customization that can significantly enhance image quality. While its operation may resemble a powerful microscope, the exoscope's advanced camera settings allow surgeons to fine-tune the image in ways that can significantly optimize the individual visual experience for each user. The increasing availability of operative visualization systems (microscopes, endoscopes, and exoscopes) has given neurosurgeons multiple options to address complex surgical pathology. Several laboratory investigations (11, 12) and clinical reports (13–15) indicate a growing adoption of exoscopic surgery.

Camera setting preferences are inherently subjective and vary according to individual user needs. For a user, the exoscope-hybrid system needs to produce accurate, pleasing, and informative images. Our analysis aimed to determine the specific impact of different camera settings on image quality, depth, and edge detection. It does not attempt to prescribe a universally ideal setting for every user. When assessing visualization technologies in surgery, the images provided by these tools must closely approximate what the surgeon would see directly. This means maintaining the integrity of visual information, like colors, depth, and texture, which is crucial for accurate tissue identification and precise surgical maneuvers. Metrics like SSIM, MSE, Laplacian filters, and depth maps allow us to assess the role of the camera settings in image distortion, edge, and depth detection relative to an unaltered exoscope image to determine how closely the digitally displayed image resembles the actual, direct view of the surgical site.

### 4.1 Exoscope does not distort images

SSIM and the MSE scores utilize mathematical algorithms that analyze image distortion relative to a reference image. SSIM assesses the similarities between pixels across different images, providing insight into how well the image details are preserved. Conversely, MSE is used to quantify the differences between pixel values in the images, effectively measuring the level of distortion introduced by various adjustments. We used a reference image (standard mode) captured by the exoscope without any modifications as a benchmark for each test. This reference image was compared against images captured with various adjustments in camera mode, aperture, contrast, sharpness, color saturation, and brilliance. The primary aim of this comparison was to determine whether different image modes and adjustments led to any significant image distortion, edge, and depth detection. By systematically comparing these images to the unaltered reference image, we could evaluate how each adjustment impacts the overall fidelity of the images, thus understanding the extent to which different settings might alter the surgical view.

Based on the SSIM scores obtained across the three different surgical approaches using various magnifications, the data reveals that the lowest SSIM score was 0.63, and the highest was 0.85. An SSIM score of −1 denotes a significant difference between images, while a score of 1 indicates that the images are identical. This means that higher SSIM scores are more similar to the reference image and have less image distortion. The range of SSIM scores in our results, all above 0, suggests minimal image distortion across the different camera settings compared to the original image. The MSE scores obtained from our study further reinforce the findings indicated by the SSIM scores. Across the three different surgical approaches at various magnifications and working distances, we observed notably low MSE scores, almost near zero. These low MSE values indicate minimal pixel-level differences between the modified and original reference images. The finding for SSIM and MSE demonstrates a high level of fidelity in the images produced under various settings, implying that the adjustments made in camera mode, aperture, contrast, sharpness, color saturation, and brilliance do not substantially distort the visual representation of the surgical site.

### 4.2 Exoscope produces sharp images

In surgical procedures that involve micro-manipulations, a sharp image with a clear definition between the anatomical target and the surrounding brain parenchyma is desirable. We used edge detection algorithms as a method to assess image sharpness. A Laplacian filter was applied to each image captured by the exoscope. This filter is an image-processing technique that can assess image sharpness and edge detection by focusing on areas where the pixel intensity changes rapidly. The variance in the filter's measurements indicates the image's high-frequency content or sharpness. Each Laplacian filter variance value is intrinsic to the analyzed image, with expected variations at different magnifications and working distances. Therefore, to determine the effectiveness of edge detection, it was necessary to assess the Laplacian filter variance and the image output from the Laplacian filter. When evaluating the Laplacian filter images, we found that the hybrid system effectively captured depth across all camera settings. The image output consistently showed edge detection even at the lowest Laplacian filter variance. This capability of the exoscope to produce sharp images and discern edges further confirms its utility in providing surgeons with high-quality images comparable to the operating microscope.

### 4.3 Exoscope captures depth of the surgical field

The depth of field captured by the exoscope camera is the critical parameter by which deep visualization of anatomical structures in different planes can be visualized. This concept applies to scenarios dealing with complex geometric relationships, such as deep cranial approaches and the optical ability to discern different depth layers of the image (16). When you make the camera's opening smaller (decrease aperture), more of the picture across different depths looks sharp. In contrast, if you make the camera's opening bigger (increase aperture), only the objects close to the camera will look sharp, while objects further away will be blurry. The relationship between depth of field, camera aperture, and focal length can be utilized to achieve superior visualization and is commonly used in other industries, such as photography (16). Traditionally, with an operative microscope, these parameters are internally adjusted and do not require the surgeon to control or enhance the optical view. However, since exoscopes utilize a camera system, depth of field and resolution can be adjusted by adjusting the camera's aperture setting (16).

To understand and analyze the impact of these camera settings on depth perception in 2D images, we employed a machine learning model trained to calculate pixel-level depth. Utilizing a vision transformer neural network (MiDaS), this model computed depth maps and histogram plots of depth allocation by recognizing depth cues within images and estimating depth allocations based on pixel frequency means and variance. We previously explored using monocular depth estimation for 3D reconstruction of neuroanatomy (17). Depth allocations were graphed as histograms from the depth maps. Histogram analysis involves plotting how many pixels (or what proportion of the image) are at each depth level. For the generated depth maps, mean pixel frequency varies with image composition, making direct comparisons challenging.

Therefore, it is essential to assess both the variance within each histogram and across histograms for different camera settings at specified magnifications and working distances to understand the consistency and accuracy of depth information. Additionally, the depth maps were visually correlated with the original image to assess the capture of deep structures. In the maps, different colors correspond to varying depths: purple indicates the detection of deeper structures, while yellow signifies more superficial ones. Carefully examining these depth maps reveals the exoscope's capability to detect deep structures accurately, regardless of the surgical approach, working distance, or magnification used. This is evident through the higher degree of purple on the depth map related to deep structures. The depth maps demonstrate the exoscope's effectiveness at capturing deep and superficial structures even when adjusting the camera settings.

### 4.4 Personal preferences, user feedback, and future directions

Our results indicate that the adjustments in camera settings, including aperture, color saturation, contrast, sharpness, and brilliance when using the exoscope, do not compromise image quality, edge, and depth detection compared to the standard setting. Surgeons can, therefore, confidently tailor the exoscope's camera settings to their preferences or the specific requirements of the clinical scenario while preserving the integrity of the image. We hope this study will encourage surgeons to familiarize themselves with the basic concepts of camera optics. Understanding camera fundamentals will help surgeons decide which visualization modality to use for a given case, especially as imaging produced by operating microscope-exoscope systems can yield a plethora of manipulations or modes that may enhance anatomical structures.

A good example is neurovascular decompression for trigeminal neuralgia, a procedure that can be performed using endoscopic, microsurgical, and exoscopic approaches (18). The endoscopic approach offers a deep view and access to narrow spaces but provides limited field depth. The microsurgical approach, on the other hand, provides a high-resolution image and depth perception but ergonomic challenges and a restricted field of view. The exoscopic approach merges the benefits of high-resolution magnification and ergonomic comfort by projecting the operative field onto a 3D monitor, allowing for a shared view with the surgical team. While the choice of operative platform ultimately depends on surgeon preference and comfort, objective metrics obtained using advanced computer algorithms can guide surgeons regarding which specific visualization platform may offer the best image, considering the target's depth and the system's optical properties.

Based on user feedback, it has been observed that narrowing the aperture tends to provide clearer images compared to widening it in high magnification settings. Conversely, widening the aperture reduces blurring around the area in low magnification settings. With a working distance of 250 mm, we could get more enlarged and sharper images than 600 mm when the zoom was maximized. There is less light loss if the distance between the object and the camera light source is short. Thus, the 250 mm working distance at maximum zoom provided a more transparent, sharper image than at 600 mm working distance. However, the disadvantage of using 250 mm was that the space between the object and the camera was narrow, making hand movement relatively uncomfortable or restricted.

In addition, users believed that the orbitozygomatic approaches yielded clearer images than the far lateral approach, supported by Table 1. The orbitozygomatic approaches involve the removal of as much of the orbit, zygoma, and sphenoid bone as necessary to minimize obstructions in the surgical field, thereby facilitating further muscle retraction. Such resection allows the exoscope's light to shine directly on the target area in the deep surgical field, improving visibility and image quality. In contrast, even after thorough condylectomy, the far lateral approach positions the light source to cast light at a relatively oblique angle. This angle can lead to increased light reflection off surrounding bony structures and muscle flaps, likely contributing to a minor decline in the clarity of digital images.

Lastly, users noted that the brilliance mode might exacerbate the operator's eye fatigue and obstruct the clear identification of anatomical structures in specific clinical scenarios. Brilliance mode produces red colors that stand out in the images. This mode allows better (i.e., more precise and sharp) observation and identification of blood vessels in the surgical field of view, and it may be advantageous to observe small or thin blood vessels more clearly. However, if surgery is performed on a patient with cerebral hemorrhage or bleeding occurs during surgery, the surgical field of view would likely be more red than usual.

### 4.5 Limitations

A limited selection of cranial exposures was included in our study and might only partially generalize across the entire spectrum of neurosurgical procedures. Other quantitative and qualitative image analysis types have been described, but we chose SSIM and MSE because of their practical implementation and broad use in different fields. The MiDaS depth model can be related to inaccuracies in detecting fine details in intraoperative images due to varying image compositions. Exploring other emerging depth models in future research could improve the accuracy of depth estimation. Our data was collected using a prototype version of a hybrid system. As the prototype is subject to further modifications and improvements, the results must be interpreted with an understanding that changes in the final version could alter the observed performance characteristics. The specific prototype system we used represents just one example of such technology, and our results may not be directly transferable to other systems. Other commercially available stand-alone exoscopic systems exist that may involve different camera optics. Future studies could compare multiple exoscopic systems, thus providing a more comprehensive evaluation of this technology in neurosurgery.

## 5 Conclusions

This study used advanced algorithms and deep learning to examine image quality, edge, and depth detection across various exoscope camera settings. We created a high-resolution image dataset examining a wide range of neurosurgical scenarios Our investigation has provided substantial evidence that adjustments in the camera settings of the exoscope did not change the integrity of image quality or introduce significant distortions when compared to the base camera setting. Using Laplacian filters and depth maps, we found that the exoscope could effectively capture information on the sharpness and depth of the surgical field. Importantly, our results should encourage surgeons to take full advantage of the exoscope's extensive range of settings, allowing them to customize the camera according to their preferences or the specific clinical situation demands. In conclusion, the exoscope is invaluable in modern surgical practice, combining high-definition imaging, ergonomic design, and adaptable operability.

## Data Availability

The raw data supporting the conclusions of this article will be made available by the authors, without undue reservation.

## References

[1] Uluc K Kujoth GC Baskaya MK. Operating microscopes: past, present, and future. Neurosurg Focus. (2009) 27(3):E4. 10.3171/2009.6.FOCUS09120 19722819

[2] Hafez A Elsharkawy A Schwartz C Muhammad S Laakso A Niemela M Comparison of conventional microscopic and exoscopic experimental bypass anastomosis: a technical analysis. World Neurosurg. (2020) 135:e293–9. 10.1016/j.wneu.2019.11.154 31805406

[3] Ricciardi L Chaichana KL Cardia A Stifano V Rossini Z Olivi A The exoscope in neurosurgery: an innovative “point of view”. A systematic review of the technical, surgical, and educational aspects. World Neurosurg. (2019) 124:136–44. 10.1016/j.wneu.2018.12.202 30660891

[4] Fiani B Jarrah R Griepp DW Adukuzhiyil J. The role of 3D exoscope systems in neurosurgery: an optical innovation. Cureus. (2021) 13(6):e15878. 10.7759/cureus.15878 34327102 PMC8302823

[5] Haeren R Hafez A Lehecka M. Visualization and maneuverability features of a robotic arm three-dimensional exoscope and operating microscope for clipping an unruptured intracranial aneurysm: video comparison and technical evaluation. Oper Neurosurg (Hagerstown). (2022) 22(1):28–34. 10.1227/ONS.0000000000000060 34982902

[6] Langer DJ White TG Schulder M Boockvar JA Labib M Lawton MT. Advances in intraoperative optics: a brief review of current exoscope platforms. Oper Neurosurg (Hagerstown). (2020) 19(1):84–93. 10.1093/ons/opz276 31529083

[7] Doron O Langer DJ Ellis JA. Exoscopic cerebrovascular neurosurgery. Neurosurg Clin N Am. (2022) 33(4):483–9. 10.1016/j.nec.2022.05.008 36229135

[8] Montemurro N Scerrati A Ricciardi L Trevisi G. The exoscope in neurosurgery: an overview of the current literature of intraoperative use in brain and spine surgery. J Clin Med. (2021) 11(1):223. 10.3390/jcm11010223 35011964 PMC8745525

[9] Smith S Kozin ED Kanumuri VV Barber SR Backous D Flavio Nogueira J Initial experience with 3-dimensional exoscope-assisted transmastoid and lateral skull base surgery. Otolaryngol Head Neck Surg. (2019) 160(2):364–7. 10.1177/0194599818816965 30598049

[10] Zhou W Bovik AC. Mean squared error: love it or leave it? A new look at signal fidelity measures. IEEE Signal Process Mag. (2009) 26(1):98–117. 10.1109/MSP.2008.930649

[11] Rindler RS Soriano RM Elsherbini MM Kim B Revuelta Barbero JM Pradilla G Exoscopic en bloc carotid-sparing total temporal bone resection: feasibility study and operative technique. World Neurosurg. (2022) 160:e1–8. 10.1016/j.wneu.2021.08.115 34481102

[12] Herlan S Marquardt JS Hirt B Tatagiba M Ebner FH. 3D exoscope system in neurosurgery-comparison of a standard operating microscope with a new 3D exoscope in the cadaver lab. Oper Neurosurg (Hagerstown). (2019) 17(5):518–24. 10.1093/ons/opz081 31140555

[13] Rosler J Georgiev S Roethe AL Chakkalakal D Acker G Dengler NF Clinical implementation of a 3D4K-exoscope (orbeye) in microneurosurgery. Neurosurg Rev. (2022) 45(1):627–35. 10.1007/s10143-021-01577-3 34142267 PMC8827320

[14] Peron S Paulli S Stefini R. Case report: high-definition 4K-3D exoscope for removal of an orbital cavernous hemangioma using a transpalpebral approach. Front Surg. (2021) 8:671423. 10.3389/fsurg.2021.671423 34422890 PMC8377276

[15] Patel NV Ligas B Gandhi S Ellis J Ortiz R Costantino P Internal maxillary to middle cerebral artery bypass using an anterior tibial artery graft, performed using a 3-dimensional exoscope: 2-dimensional operative video. Oper Neurosurg (Hagerstown). (2020) 19(2):E187. 10.1093/ons/opz379 31811302

[16] Strong S. Introduction to Visual Optics. 6th edn. Amsterdam: Elsevier (2023).

[17] Gonzalez-Romo NI Hanalioglu S Mignucci-Jimenez G Abramov I Xu Y Preul MC. Anatomic depth estimation and 3-dimensional reconstruction of microsurgical anatomy using monoscopic high-definition photogrammetry and machine learning. Oper Neurosurg (Hagerstown). (2023) 24(4):432–44. 10.1227/ons.0000000000000544 36701667

[18] Nagata Y Watanabe T Nagatani T Takeuchi K Chu J Wakabayashi T. The multiscope technique for microvascular decompression. World Neurosurg. (2017) 103:310–4. 10.1016/j.wneu.2017.04.059 28434953

